# The role of gut microbiota and blood metabolites in postpartum depression: a Mendelian randomization analysis

**DOI:** 10.3389/fcimb.2024.1416298

**Published:** 2024-07-10

**Authors:** Ji Cui, Qilong Zhai, Zhu Yang, Yi Liu

**Affiliations:** ^1^ Department of Obstetrics and Gynecology, The Second Affiliated Hospital of Chongqing Medical University, Chongqing, China; ^2^ Department of Hepatobiliary Surgery, The Second Affiliated Hospital of Chongqing Medical University, Chongqing, China

**Keywords:** postpartum depression, gut microbiota, metabolism, Mendelian randomization, GWAS, causality

## Abstract

**Background:**

Postpartum depression (PPD) is a common complication of pregnancy that imposes a heavy health and economic burden on individuals, families and society. The etiology of PPD is complex and incompletely defined, and recent studies have identified an important role for gut microbiota (GM) and their metabolites in neurological disorders. However, fewer studies on GM and PPD are available and have not yielded uniform results.

**Methods:**

Instrumental variables for GM and blood metabolites were obtained from the MiBioGen consortium and metabolomics GWAS server. Single nucleotide polymorphisms (SNPs) associated with PPD phenotypes were obtained from the FinnGen consortium. Inverse variance weighted (IVW), weighted median, weighted mode, and MR-Egger methods were used to assess causal effects. Inverse MR analysis and sensitivity analysis were also utilized to improve the stability of the results.

**Results:**

In this study, 5 intestinal species and 24 blood metabolites causally associated with PPD were identified using MR analysis. In addition, MR analysis showed that *Prevotellaceae and Bifidobacteria* may reduce the risk of PPD by elevating Xanthine and 1-arachidonoylglycerophosphoinositol (LysoPI) levels.

**Conclusions:**

This study identified GM and blood metabolites causally associated with PPD. The results of this study may provide a theoretical basis for the discovery of PPD-related biomarkers and the treatment of the disease by regulating the gut microenvironment.

## 1 Introduction

Postpartum depression (PPD) is a form of depression that manifests between four weeks and twelve months following childbirth, representing a prevalent complication among postpartum women (O’Hara and McCabe, 2013). The clinical presentations and diagnostic criteria for PPD align with those of non-perinatal depression, primarily characterized by sadness, despondency, crying, irritability, and in severe instances, symptoms of psychosis such as hallucinations and suicidal ideation (Pearlstein et al., 2009). Notably, PPD patients often exhibit an excessive concern for their infant’s health and safety. Suicidal ideation is notably prevalent in PPD sufferers, with research indicating that approximately 20% entertain thoughts of self-harm, occasionally extending these harmful thoughts towards the infant (Stewart and Vigod, 2016). PPD remains significantly under-recognized and under-valued in societal discourse, despite evidence suggesting that up to 70% of expectant mothers experience mild depressive symptoms or episodes during pregnancy (Payne, 2021), with a subset subsequently diagnosed with PPD. Globally, PPD affects 10-15% of new mothers, with incidence rates varying across regions based on healthcare and socioeconomic conditions, generally higher in low- to middle-income countries (Forty et al., 2006; Howard et al., 2014).

PPD entails profound ramifications for mothers, infants, and their partners. It has been identified as the second most common cause of mortality among postpartum women, attributable to suicide (Peay, 2020). Moreover, PPD detrimentally influences caregiving practices, including breastfeeding and mother-infant interactions, potentially compromising the infant’s emotional and cognitive development. Chronic depression in women is associated with diminished language abilities and reduced activity levels, which may escalate familial conflicts and disrupt family harmony (Goodman et al., 2011). A 2021 study highlighted that individuals with PPD incur notably higher childbirth-related expenses, experience greater birth morbidity, and face increased hospitalization costs compared to those unaffected by PPD (Brown et al., 2021). Consequently, PPD places a considerable emotional and financial strain on individuals, families, and the broader society.

The risk factors contributing to PPD are multifaceted, with a significant emphasis on psychosocial elements and genetic predispositions in current research. Women experiencing PPD often exhibit heightened anxiety levels, endure less satisfactory marital relationships, and possess weaker social connections compared to those undergoing typical childbirth (Robertson et al., 2004). Furthermore, a familial history of PPD, particularly among mothers and sisters, markedly increases the risk of its occurrence (Forty et al., 2006). The underlying mechanisms of PPD remain intricate and elusive, with hormonal fluctuations, particularly in reproductive hormones, being implicated in its onset. Preliminary findings from a limited study suggest that estradiol treatment may alleviate symptoms in PPD sufferers (Gregoire et al., 1996). Additionally, genetic and immunological factors are believed to play roles in PPD, though comprehensive studies yielding conclusive results are scarce.

Treatment modalities for PPD remain constrained. Traditional approaches encompass maternal care strategies, psychological therapies, and augmented social support networks. In cases of severe PPD, the administration of antidepressants, specifically Selective Serotonin Reuptake Inhibitors (SSRIs), becomes imperative. Although a majority of instances show improvement within months following intervention, approximately 24% and 13% of individuals diagnosed with PPD continue to experience depressive symptoms one and two years post-delivery, respectively (Sohr-Preston and Scaramella, 2006). This underscores the critical need for the identification and development of novel, safe, and efficacious treatments for PPD. The role of gut microbiota (GM) has emerged as a significant area of interest in recent research, particularly concerning its influence on the gut-brain axis and its potential implications in the etiology and progression of neuropsychiatric disorders (Wang et al., 2023). Notably, the substantial alterations observed in GM during the postpartum period highlight its potential as a pivotal factor in PPD pathogenesis (Simpson et al., 2021).

The human GM is very diverse and numerous, with a genome more than 100 times larger than the human genome (Yu et al., 2019). This intricate and symbiotic co-evolution with the host underscores the GM’s pivotal role in biological functions, contingent upon its compositional and quantitative attributes. Observational research has underscored notable disparities in the GM profiles of pregnant women with PPD compared to those without (Cheung et al., 2019). Additionally, metabolites produced by the GM, including short-chain fatty acids (SCFA), catecholamines, histamine, and γ-aminobutyric acid (GABA), are known to influence brain metabolism and functionality, either directly or indirectly, suggesting a significant regulatory impact of the GM on PPD homeostasis (Liu et al., 2020). Nonetheless, due to ethical considerations, research exploring the GM-PPD nexus remains largely observational. Such studies are inherently vulnerable to potential biases, including confounding variables and reverse causation, which obfuscate the elucidation of a definitive causal link.

Recent advancements in genomics and high-throughput sequencing technologies have significantly augmented our ability to scrutinize the GM. There is an expanding corpus of research exploring the intricate link between the human microbiota and a spectrum of female reproductive system disorders. Among these, Mendelian Randomization (MR) analysis stands out as a cutting-edge technique that addresses the constraints of conventional research methodologies (Birney, 2022). By utilizing genetic variations associated with specific exposures as instrumental variables (IVs), MR analysis aims to elucidate the causal relationships between modifiable exposures and their effects. The stochastic nature of genetic variant allocation at conception allows MR to sidestep issues like confounding factors and reverse causation, presenting a robust alternative to traditional studies. This unique methodological strength positions MR analysis as a more dependable approach to ascertain causality. In this context, MR analysis was applied to dissect the relationship between GM, blood metabolites and PPD, and to decipher the influence of GM on PPD via metabolic pathways, thereby shedding light on underlying biological mechanisms.

## 2 Methods

### 2.1 Data sources

#### 2.1.1 GWAS for GM

This research utilized the GM Genome-wide association study (GWAS) dataset from the MiBioGen consortium’s extensive meta-analysis, which stands as the most comprehensive endeavor to date in mapping microbiota composition (Kurilshikov et al., 2021). The MiBioGen initiative, focused on elucidating the interplay between host genetics and microbiome composition, orchestrated the largest multi-ethnic genome-wide investigation in this domain. The study encompassed 18,340 participants from 24 distinct cohorts, including 13,266 individuals of European ancestry. The analysis identified 122,110 genetic loci associated with the presence of 211 bacterial taxa within the gut microbiome, covering 131 genera, 35 families, 20 orders, 16 classes, and 9 phyla.

#### 2.1.2 GWAS for PPD

The genomic analysis in this study drew upon the GWAS summary statistics for PPD sourced from the FinnGen Consortium’s R6 release data, which was made accessible to the public in January 2022. This particular dataset comprised a cohort of 78,633 participants, including 9,392 individuals identified as cases of PPD and 69,241 controls. PPD is characterized as a depressive episode occurring within the timeframe of four weeks to twelve months following childbirth. Additional details regarding the study and its findings can be accessed via the FinnGen project’s website (https://www.finngen.fi/en/access_results).

#### 2.1.3 GWAS for blood metabolites

The genetic underpinnings of blood metabolites were explored using data from the metabolomics GWAS server (https://metabolomics.helmholtz-muenchen.de/gwas/). In a comprehensive analysis, Shin et al. successfully mapped nearly 2.1 million Single Nucleotide Polymorphisms (SNPs) to 486 metabolites linked to human genetic variations through Genome-wide association studies combined with high-throughput metabolic profiling (Shin et al., 2014). This investigation encompassed 7,824 individuals of European ancestry, incorporating 1,768 participants from the KORA F4 study in Germany and 6,056 from the UK Twin Study. Of the scrutinized metabolites, 107 remained unidentified due to their yet-to-be-elucidated chemical properties. The remaining 309 metabolites were chemically verified and classified into eight broad categories of metabolic pathways: amino acids, carbohydrates, cofactors and vitamins, energy, lipids, nucleotides, peptides, and xenobiotic metabolism.

GWAS summary-level data used in this study was shown in **Table 1**.

**Table 1:** GWAS summary-level data used in this study.

Phenotype	Consortium	Participants	Case	Control	Ancestry
Gut microbiota	MiBioGen	18,340	/	/	Multiracial
Postpartum depression	FinnGen	78,633	9,392	69,241	European
Blood metabolites	Shin et al.	7,824	/	/	European

### 2.2 Instrumental variable (IV) selection

To guarantee the integrity and dependability of the MR analysis conducted in this study, stringent quality control measures were implemented for the selection of SNPs as IVs, in alignment with the three core principles underpinning MR. Initially, the study established a significance threshold for gene loci associated with the GM at P < 1 × 10^-5^, and SNPs exhibiting linkage disequilibrium (LD) were excluded (r^2^ ≥ 0.001 within a clumping window of 10,000 kb), following the methodology outlined by Sanna et al (Sanna et al., 2019). The details concerning the IVs selected for this analysis are presented in **Supplementary Table 1**. Furthermore, the robustness of the IVs was evaluated using the F-statistic, with genetic instruments demonstrating F-statistics below 10 being omitted from the MR analysis to ensure the strength and reliability of the instrumental variables employed (Noyce et al., 2017).

### 2.3 Statistical analysis

To uphold the integrity of the results, this study was meticulously designed to conform to strict assumptions. This included establishing a strong correlation between the instrumental variables (IVs) and the exposure variables under scrutiny, while simultaneously ensuring that the IVs bore no direct relation to the outcomes being examined (Richmond and Davey Smith, 2022). Moreover, it was imperative that the IVs were not influenced by confounding factors, which could potentially distort the findings of the study.

This study employed four diverse MR methodologies to assess the causal impact of GM and blood metabolites on PPD, each method incorporating specific assumptions about horizontal pleiotropy. The inverse variance weighted (IVW) approach, which deduces causality through a meta-analysis of Wald estimates, was designated as the primary method of analysis (Hartwig et al., 2017).

**Figure 1:**
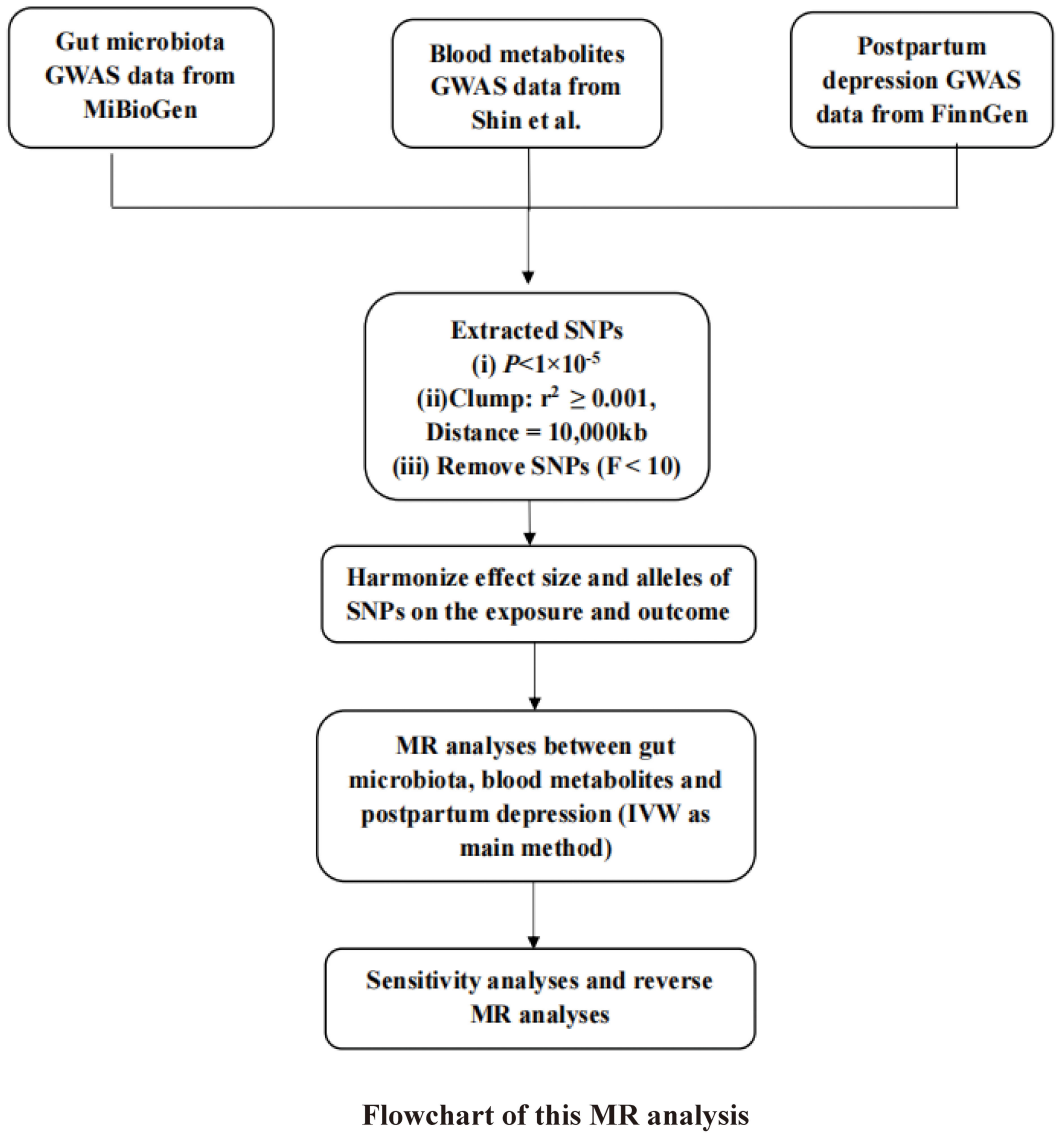
The basic assumptions of MR and study design.

To further validate the inferred causal connection between GM and PPD, the study conducted a reverse MR analysis. In this analysis, GWAS data related to PPD were treated as the exposure variables (with a significance threshold of P < 1.0×10^-5^), and the causative bacterial genera were considered the outcomes. The procedural steps and methodologies of the MR analysis are elucidated in a flowchart depicted in **Figure 1**, providing a clear visual representation of the study’s analytical framework.

### 2.4 Sensitivity analyses

To ensure the reliability of its conclusions, the study implemented a range of sensitivity analyses. Cochran’s Q test was applied to assess heterogeneity among SNPs associated with each microbial species, examining the consistency of the instrumental variables’ effects. The impact of individual SNPs on the overall causal inference was scrutinized through a leave-one-out analysis, identifying any instrumental variables that disproportionately influenced the results (Chen et al., 2021). Additionally, to evaluate the presence of horizontal pleiotropy, which could potentially bias the causal estimates, both MR-Egger regression and the MR pleiotropy residual sum and outlier (MR-PRESSO) test were employed (Ong and MacGregor, 2019). These comprehensive analyses, coupled with data visualization techniques, were conducted utilizing the “TwoSampleMR” and “MRPRESSO” packages within the R software environment, version 4.2.0 (Hemani et al., 2018).

## 3 Results

### 3.1 Selection of instrument variables

Following rigorous quality control measures, 2,033 SNPs were selected as instrumental variables for 211 intestinal species and 4,073 SNPs were selected as instrumental variables for 486 blood metabolites. Each SNP associated with GM and blood metabolites demonstrated F-statistics greater than 10, indicating a low probability of results being compromised by weak instrument bias (**Supplementary Table 1**).

### 3.2 Data analysis

#### 3.2.1 GM and PPD

**Figure 2:**
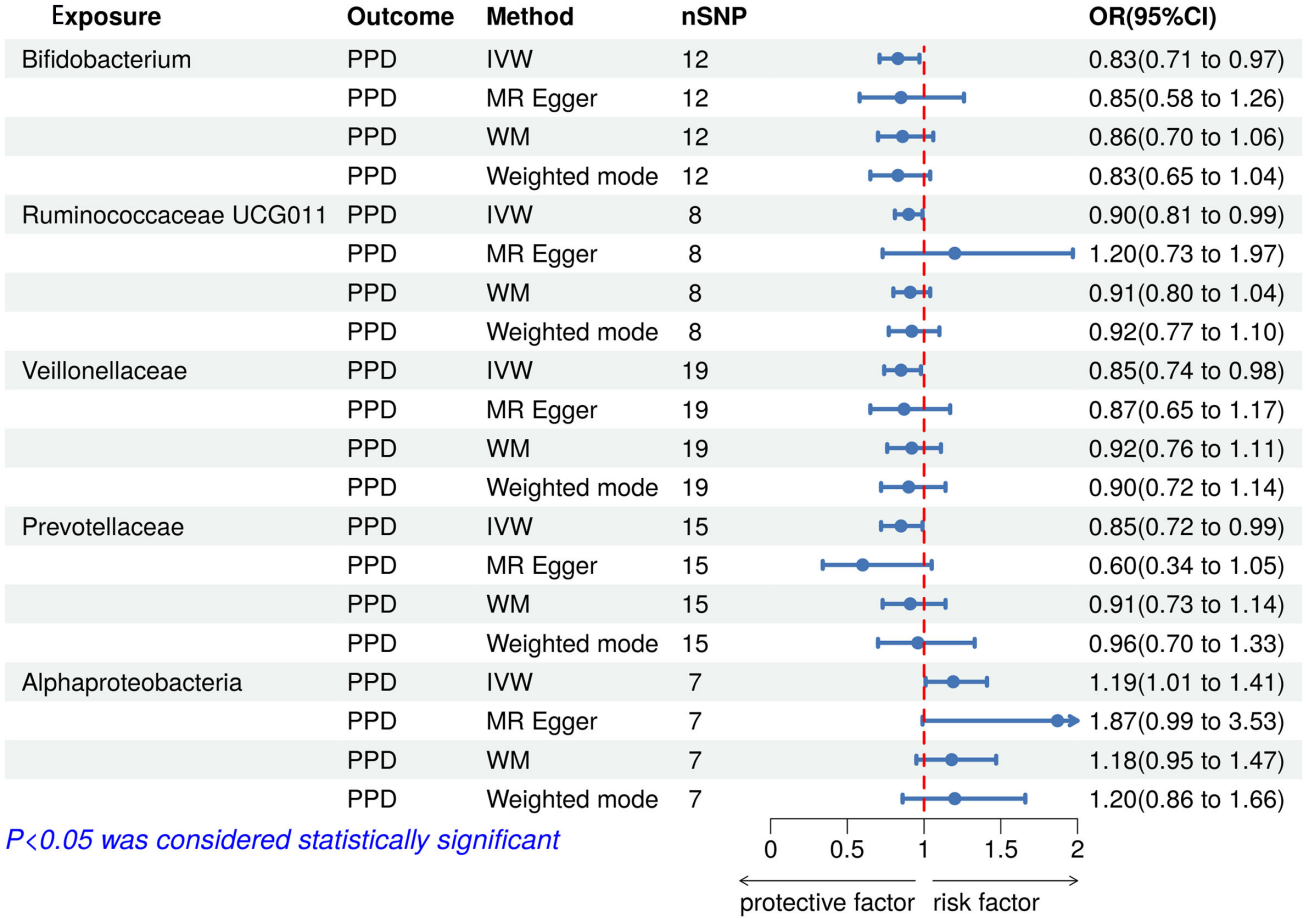
Forest plot of the associations between genetically determined gut microbial taxa with the risks of PPD. PPD, Postpartum depression; SNP, Single nucleotide polymorphisms; IVW, Inverse variance weighted; WM, Weighted median; OR, Odd ratio.

**Figure 3:**
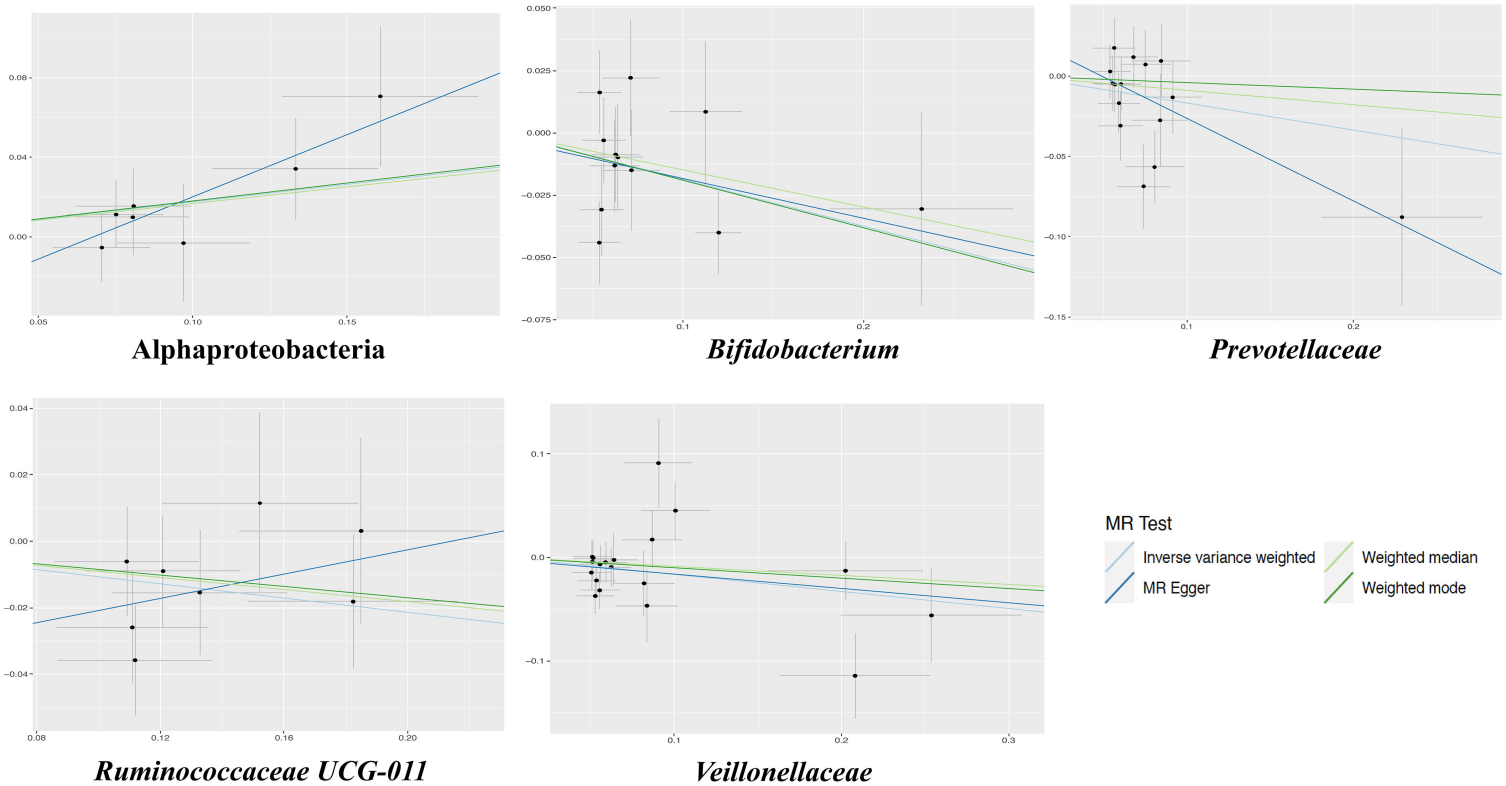
The scatter plots for the causal association between gut microbial taxa and PPD.

**Figure 4:**
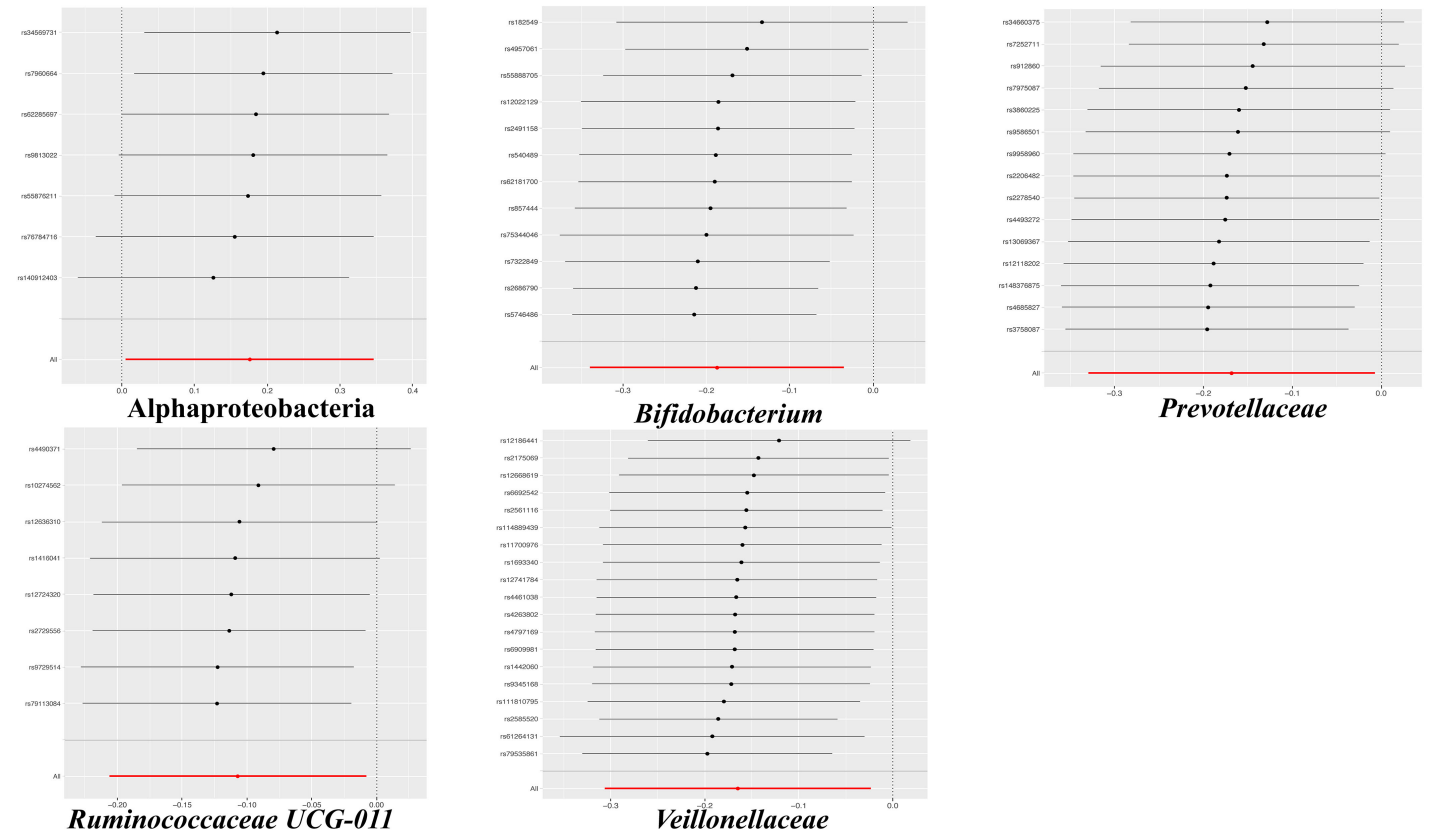
The leave-one-out plots for the causal association between gut microbial taxa and PPD.

The results of the IVW test indicated that the genetically predicted 5 intestinal species exhibited significant associations with increased or decreased risk of PPD (P < 0.05). At the genus level, MR analysis revealed a relation between the decreased risk of PPD and the higher relative abundance of *Bifidobacterium* (OR = 0.83, 95% CI: 0.71-0.97, P = 0.02) and *Ruminococcaceae UCG011* (OR = 0.90, 95% CI: 0.81-0.99, P = 0.03) as predicted genetically. At the family level, genetically predicted *Veillonellaceae* (OR = 0.85, 95% CI: 0.74-0.98, P = 0.02) and *Prevotellaceae* (OR = 0.85, 95% CI: 0.72-0.99, P = 0.04) high abundance were positively linked to a decreased risk of PPD. In addition, at the class level, this research found a strong causal association between Alphaproteobacteria (OR = 1.20, 95% CI: 1.01-1.41, P = 0.04) with a decreased risk of PPD. The association details between GM and PPD are documented in **Supplementary Table 2** and **Figure 2**. Scatter plots in **Figure 3** displayed the causal relationship between *Bifidobacterium, Ruminococcaceae UCG011*, *Veillonellaceae*, *Prevotellaceae* and Alphaproteobacteria with PPD across four MR methods. Sensitivity analyses, including Cochran’s Q test, MR-Egger, and MR-PRESSO (**Supplementary Table 3**), found no indication of pleiotropy (P > 0.05), and the Leave-one-out analysis didn’t identify any outlier SNPs, suggesting the robustness of the causal relationships without any single SNP bias (**Figure 4**). In the reverse MR analysis, no evidence of a causal effect of PPD on the aforementioned microbial taxa was observed. This suggests that the results of our MR analyses are not affected by reverse causation.

#### 3.2.2 Blood metabolites and PPD

**Figure 5:**
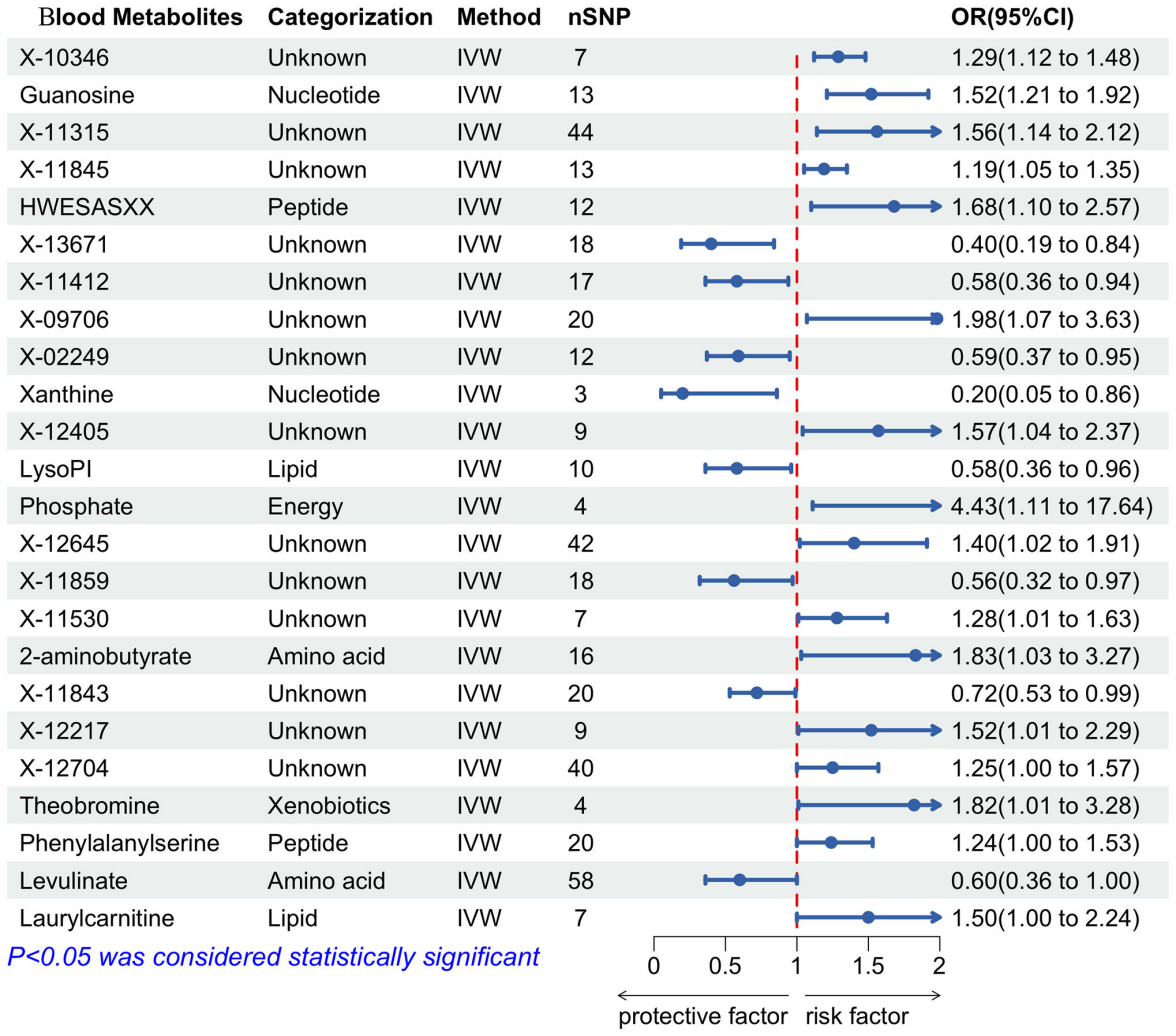
Forest plot of the associations between blood metabolites with the risks of PPD. SNP, Single nucleotide polymorphisms; IVW, Inverse variance weighted; OR, Odd ratio.

IVW analysis was performed to preliminarily identify 24 metabolites with potential causal effects on PPD, including 10 metabolites whose chemical identity is known and 14 metabolites whose chemical identity is unknown. As shown in **Figure 5**, the 10 known metabolites were chemically assigned to the amino acid, energy, lipid, nucleotide, peptide, and xenobiotics. After combining complementary and sensitivity analyses, 10 eligible metabolites that met the rigorous screening criteria were identified as candidates, including guanosine (OR = 1.52, 95% CI: 1.21-1.92, P = 3.69×10^-4^), hwesasxx (OR = 1.68, 95% CI: 1.10-2.57, P = 0.02), 1-arachidonoylglycerophosphoinositol (LysoPI) (OR = 0.58, 95% CI: 0.36-0.96, P = 0.03), phosphate (OR = 4.43, 95% CI: 1.11-17.64, P = 0.03), 2-aminobutyrate (OR = 1.83, 95% CI: 1.03-3.27, P = 0.04), xanthine (OR = 0.20, 95% CI: 0.05-0.86, P = 0.03), theobromine (OR = 1.82, 95% CI: 1.01-3.28, P = 0.05), phenylalanylserine (OR = 1.24, 95% CI: 1.00-1.53, P = 0.05), levulinate (OR = 0.60, 95% CI: 0.36-1.00, P = 0.05), laurylcarnitine (OR = 1.50, 95% CI: 1.00-2.24, P = 0.05). In summary, the estimates derived from IVW are significant (p < 0.05) and the Cochran Q test (p > 0.05) and MR-Egger intercept test (p > 0.05) provided strong evidence for the absence of heterogeneity and pleiotropy (**Supplementary Table 4**). LOO analysis results supported that a single SNP did not cause bias in MR estimation. These eleven blood metabolites are considered to be candidates for further analysis.

#### 3.2.3 GM and blood metabolites

To further investigate whether GM can influence the development of PPD by affecting metabolic pathways. We performed MR analysis of five identified known intestinal species and ten blood metabolites that are causally associated with PPD (**Supplementary Table 5**). MR analysis showed no significant causal relationship between the three intestinal species (*Ruminococcaceae UCG011*, *Veillonellaceae* and Alphaproteobacteria) and blood metabolites. In MR analysis, IVW method showed a causal relationship between *Prevotellaceae* and Xanthine (OR = 1.04, 95% CI: 1.01-1.08, P = 0.02). Additionally, the WM method showed a causal relationship between *Bifidobacterium* and LysoPI (OR = 1.24, 95% CI: 1.00-1.53, P = 0.05). The IVW method also showed a statistically significant causal relationship between *Bifidobacterium* and LysoPI as well (OR = 1.07, 95% CI: 1.01-1.14, P = 0.03), when the screening threshold for intestinal flora was raised to P<5.0×10^-6^. This suggested that *Prevotellaceae* and *Bifidobacterium* may have a protective effect on PPD by elevating Xanthine and LysoPI levels.

## 4 Discussion

**Figure 6:**
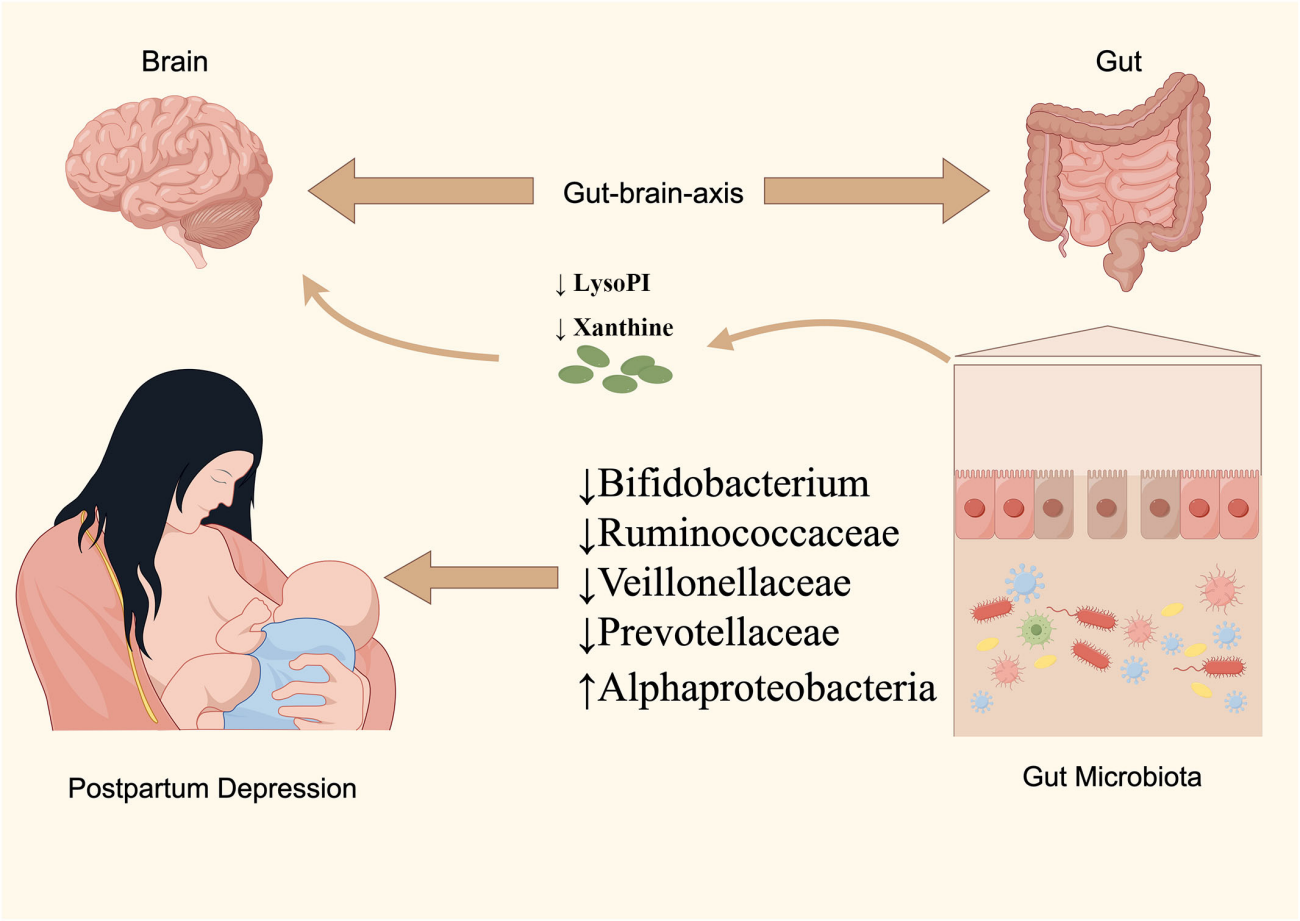
The illustration represents significant causal gut microbial taxa associated with PPD.

The etiology of PPD remains partially understood, with contemporary research linking its development to neurofunctional disruptions, the hypothalamic-pituitary-adrenal (HPA) axis, immune responses, and the gut-brain axis dynamics (Chaudhury et al., 2015). The bidirectional regulatory mechanism of the gut-brain axis, highlighted in recent studies, suggests the nervous system’s influence on gut functionality and balance, as well as the impact of GM and their metabolites on the autonomic and neuroendocrine systems (Rackers et al., 2018). Consequently, the established causal linkage between GM and mental health disorders via the gut-brain axis has garnered increasing scholarly attention. Notably, a meta-analysis by Sanada K et al. revealed significant variations in gut microorganisms between depressed patients and controls, with similar patterns observed in PPD subjects in this research (Sanada et al., 2020). We identified five gut species with a causal relationship to PPD; specifically, Alphaproteobacteria was linked to a heightened risk, whereas *Bifidobacterium*, *Veillonellaceae*, *Ruminococcaceae UCG-011*, and *Prevotellaceae* were associated with a diminished risk. Moreover, our findings implicate various metabolic pathways, including those involved in energy, amino acids, steroids, and neurotransmitters, in PPD’s pathogenesis. Utilizing MR techniques, this study identified 10 known blood metabolites causally related to PPD, primarily involving nucleotides, peptides, energy substrates, xenobiotics, amino acids, and lipids, positioning them as potential metabolic markers for PPD. Interestingly, this research implies that *Prevotellaceae* and *Bifidobacterium* may play a regulatory role in the development of PPD through metabolic pathways, a novel insight not previously reported (**Figure 6**).

Emerging research indicates that the GM plays a pivotal role in the onset of PPD by influencing various pathways, including those involving reproductive hormones, serotonin (5-HT), SCFA, amino acids, and the HPA axis (Cheung et al., 2019). Specifically, the dramatic hormonal fluctuations characteristic of pregnancy and the postpartum period, such as changes in estrogen and luteinizing hormone levels, are linked to PPD. These hormonal shifts may be exacerbated by GM disturbances post-delivery, which affect hormone-regulating enzymes, subsequently impacting the GABA and dopaminergic systems and contributing to PPD development (Simpson et al., 2021). Central to depression’s pathophysiology, 5-HT’s synthesis and regulation are influenced by various microorganisms, including Streptococcus and Escherichia (O’Mahony et al., 2015). Animal models have demonstrated that GM can mitigate depressive-like behaviors by enhancing 5-HT, glucagon-like peptide-1 (GLP-1), and GLP-1 receptor concentrations, alongside upregulating brain-derived neurotrophic factor (BDNF) expression (Sun et al., 2018). As a key GM metabolite and gut-brain axis mediator, SCFA has been implicated in depressive manifestations, potentially exerting antidepressant effects through inflammatory response reduction and neurotransmitter synthesis enzyme activity modulation. Studies by Pu J et al. revealed significant alterations in plasma amino acid levels in depression patients compared to healthy controls, with these changes linked to gut microbial metabolism, highlighting a potential pathogenic mechanism of GM-induced depression (Pu et al., 2021). Certain amino acids and their bacterial-derived metabolites have been proposed as PPD biomarkers. Furthermore, the HPA axis’s role in PPD is underscored by its stimulation of corticotropin-releasing hormone (CRH) and adrenocorticotropic hormone (ACTH) release, leading to cortisol production. Fluctuations in cortisol and CRH levels are implicated in PPD, with cortisol influencing GM composition and, conversely, GM modulating HPA axis activity through cortisol regulation, suggesting a bidirectional regulatory mechanism between GM and the HPA axis (Sudo et al., 2004).

At the genus level, this study corroborated the findings of Bosch et al., indicating that *Ruminococcaceae UCG011* is linked to a diminished risk of developing PPD (Bosch et al., 2022). This genus, part of the *Ruminococcaceae* family, consists of bacteria capable of breaking down dietary fibers into SCFAs such as acetic, propionic, and butyric acids. Prior research, including PPD mouse models, has highlighted the potential of increased dietary fiber and consequent SCFA levels to mitigate depressive behaviors (Liu et al., 2020). Specifically, butyrate has been shown to possess antidepressant-like effects by enhancing 5-HT levels in the brain, boosting BDNF expression, and repairing blood-brain barrier (BBB) integrity. Similarly, our findings suggest a protective role of *Bifidobacterium* against PPD, aligning with Aizawa, E et al.’s observation of reduced *Bifidobacteria* and *Lactobacillus* in Major Depressive Disorder (MDD) patients (Aizawa et al., 2016). *Bifidobacterium*, constituting 3-5% of the adult gut microbiome, is increasingly recognized for its therapeutic potential in mental health, supported by evidence from a randomized clinical trial where *Bifidobacterium breve CCFM1025* alleviated depression by modulating GM and tryptophan metabolism (Tian et al., 2022). The exact mechanisms remain to be fully elucidated, though studies suggest *Bifidobacterium*’s ability to modulate the HPA axis and mitigate depression-like symptoms via the serotonin reuptake pathway (Sudo et al., 2004). Our research also identified a novel association between *Bifidobacterium* and elevated levels of LysoPI, a lysophosphatidylinositol potentially implicated in immune and inflammatory responses, as suggested by its links to conditions like asthma, COVID-19, and cervical inflammation in existing literature (Zhang et al., 2018; Gu et al., 2022; Tu et al., 2024). Furthermore, the relevance of phospholipid metabolism, particularly LysoPI, in PPD pathophysiology, underscores the potential of dietary interventions, such as ϵ-polylysine supplementation, to influence gut microbiota and blood metabolite profiles, offering promising avenues for PPD prevention and treatment (Zhang et al., 2022).

At the family level, this study discovered that *Prevotellaceae* and *Veillonellaceae* are inversely associated with the risk of PPD, corroborating findings from earlier observational research (Barandouzi et al., 2020). *Prevotellaceae*, known for its SCFA production, appears to mitigate depressive symptoms through anti-inflammatory mechanisms, notably by activating G protein-coupled receptors (GPCRs) and inhibiting histone deacetylases (HDACs) in intestinal epithelial and immune cells, thereby improving depressive behaviors. Interestingly, this study used MR analysis to find that Prevotellaceae appears to reduce the risk of PPD by elevating Xanthine levels. This is a pathway of action of gut flora not reported in previous studies. The relationship between Xanthines and PPD is still understudied and the exact mechanism is not clear. In depressed patients, Xanthine levels are higher than in the normal population, however, MR analyses have not yielded consistent conclusions, which may be due to the specific mechanism of action of Xanthines in different types of depression. Therefore, further studies are still needed to clarify the mechanism of action of this same pathway. On the other hand, the relationship between *Veillonellaceae* and PPD is less explored, although existing literature suggests its association with the remission or stabilization of immune-mediated conditions such as asthma and autism. Given the involvement of immune factors in PPD, it is postulated that *Veillonellaceae* may offer protection against PPD by modulating immune responses (Parada Venegas et al., 2019).

At the class level, this research identified a significant causal link between the class Alphaproteobacteria and an increased risk of PPD. This finding aligns with the work of Mihai S Cirstea et al., which also reported a notable positive association between the abundance of Alphaproteobacteria colonies and depression levels (Cirstea et al., 2022). Alphaproteobacteria, a class within the Proteobacteria phylum, predominantly consists of gram-negative bacteria. Despite this correlation, the relationship between Alphaproteobacteria and PPD remains under-explored, and the underlying mechanisms are yet to be fully understood. One hypothesis suggests that this association may stem from Alphaproteobacteria’s potential to amplify inflammation and immune responses, possibly through lipopolysaccharide (LPS) activity, a theory that warrants further investigation.

Pu J et al.’s research demonstrated significant differences in plasma amino acid concentrations between depressed individuals and healthy controls, suggesting an association with gut microbial metabolism, a recognized contributor to depression’s pathogenesis (Pu et al., 2021). Consistent with these findings, our study identified 10 blood metabolites as potential biomarkers for PPD, highlighting their involvement in PPD’s development. Specifically, phosphate and 2-aminobutyrate were acknowledged for their critical roles in PPD by various studies. Moreover, this research established a causal link between PPD and additional metabolites such as guanosine, theobromine, laurylcarnitine, xanthine, and levulinate, previously associated with depression. These insights lay the groundwork for understanding the metabolic underpinnings of PPD, although further experimental validation is required.

This study is the first to explore the causal relationship between GM, blood metabolites and PPD using MR methods. It must be acknowledged that there are some deficiencies in this research. (i) A lenient threshold (P < 1 × 10^-5^) was applied to ensure an adequate number of IVs, potentially including weaker instruments. (ii) The GWAS data primarily involved European populations, limiting the applicability of the results to diverse ethnic groups. (iii) Gut microbial taxa were identified using three specific regions of the 16S rRNA gene, analyzing only at the genus level, not extending to species or strains. Future studies employing shotgun metagenomic sequencing could offer more precise insights. (iv) This study used GM data including multi-ethnic male and female participants, whereas GWAS data on PPD was conducted for female Europeans. But it is worth noting that the SNPs included in the study did not involve the sex chromosomes, which further minimized the influence of the gender factor on the results. (v) Age-related stratification was not possible, so the impact of gut flora at different ages could not be further determined. Further experimental and clinical studies to validate the relationship between GM, blood metabolites and PPD are extremely important.

In summary, PPD represents a prevalent complication of childbirth with extensive adverse impacts on individuals, families, and the broader community. This research successfully identified 5 intestinal species and 10 blood metabolites with causal links to PPD through MR analysis. The findings offer a foundational theoretical framework for identifying biomarkers associated with PPD and suggest potential therapeutic interventions through modulation of the gut microenvironment.

## Data availability statement

The original contributions presented in the study are included in the article/**Supplementary Material**. Further inquiries can be directed to the corresponding author.

## Ethics statement

Ethical approval and consent to participate in the original genome-wide studies (GWAS) were obtained from relevant review boards. The current study used publicly available summary statistics data, and thus no additional ethics approval was required.

## Author contributions

JC: Conceptualization, Data curation, Formal analysis, Investigation, Methodology, Resources, Software, Validation, Visualization, Writing – original draft. QZ: Data curation, Methodology, Software, Validation, Writing – original draft. ZY: Project administration, Supervision, Writing – review & editing. YL: Funding acquisition, Project administration, Supervision, Writing – review & editing.

## Supplementary material

Supplementary Table 1 The instrumental variables used in this MR analysis.

Supplementary Table 2 The result of MR analysis between gut microbiota and PPD.

Supplementary Table 3 The result of sensitivity analysis between gut microbiota and PPD.

Supplementary Table 4 The result of MR analysis between blood metabolites and PPD.

Supplementary Table 5 MR analysis of five identified known intestinal species and ten blood metabolites.
